# Development of Langat virus infectious clones as a platform for live-attenuated tick-borne encephalitis vaccine

**DOI:** 10.1038/s44298-025-00129-6

**Published:** 2025-05-23

**Authors:** Naveed Asghar, Rita Jaafar, Anna Valko, Olivia Merinder, Karl Ljungberg, Carl Mårten Lindqvist, Magnus Johansson

**Affiliations:** 1 School of Medical Sciences, Faculty of Medicine and Health, Örebro University, Örebro, Sweden; 2 Scantox Sweden, Solna, Sweden; 3 International Vaccine Institute, Europe Regional Office, Solna, Sweden; 4 Clinical Genomics, Faculty of Medicine and Health, Örebro University, Örebro, Sweden

**Keywords:** Live attenuated vaccines, Virology

## Abstract

Tick-borne encephalitis (TBE) is one of the most important tick-transmitted diseases in Europe and Asia. With no specific antiviral treatment available, vaccination remains the most effective protective strategy for TBE. Unlike currently available inactivated TBE vaccines that require repeated boosters, live-attenuated vaccines could offer lifelong immunity with a single dose. Langat virus (LGTV) is a naturally attenuated strain of TBE virus (TBEV). In this study, we engineered and rescued four infectious clones (ICs) of LGTV using RNA- and DNA-based reverse genetics methods. The ICs rescued by DNA-based method showed higher genetic stability in cell culture. One of the ICs rescued by DNA-based method was further evaluated in vitro and in vivo, which exhibited growth kinetics and immune profile comparable to the LGTV strain in our laboratory. This reverse genetics platform will be utilized to introduce targeted mutations within the LGTV genome to develop a live-attenuated TBE vaccine.

## Introduction

Tick-borne encephalitis (TBE), caused by TBE virus (TBEV), is one of the most important tick-borne diseases in Europe and Asia, with clinical manifestations ranging from mild flu-like symptoms to severe neurological disorders^1^. Over the last decades, the incidence of TBE has increased dramatically in several European countries, including Sweden^2,3^. Despite its increasing prevalence, there is currently no antiviral treatment available for TBE, making vaccination the most effective protective measure^4^.

The commercially available TBE vaccines are based on inactivated TBEV particles. Despite being effective, these inactivated vaccines require regular booster doses to sustain immunity and occasional vaccine breakthrough cases have been reported, mainly in the elderly^5^. In contrast, live-attenuated orthoflavivirus vaccines have the potential to provide lifelong immunity after a single dose because they mimic natural viral infection, stimulating both innate and adaptive immunity, thus triggering a robust immune response through neutralizing antibodies and T cell responses^6^. Langat virus (LGTV) is a naturally attenuated strain of TBEV and a potential candidate for a live-attenuated TBE vaccine. An attenuated variant of LGTV, strain TP21 was previously evaluated in human clinical trials in the 1970s in Russia. Despite its high potential, the trial was discontinued after appearance of significant adverse neurological symptoms in 35 out of 649,470 vaccinees, as reviewed by Gritsun et al.^7^. Efforts have previously been made to develop a live-attenuated TBE vaccine using LGTV or various TBEV strains^8^. Several attempts were made to further attenuate the LGTV strain TP21 by serial passaging in chicken embryo cells, use of mutagen, or production of chimeric LGTVs^9–11^.

Orthoflaviviruses like TBEV and LGTV contain a positive-sense single-stranded RNA genome that translates into a single polyprotein. This polyprotein is further processed into three structural proteins and seven non-structural (NS) proteins. The NS proteins are essential for the orthoflavivirus replication cycle to produce additional copies of the viral genome. The newly synthesized genome is then encapsidated by the structural proteins to form virions. In this study, we harnessed the natural cellular events to initiate the orthoflavivirus replication cycle leading to the production of LGTV infectious clones (ICs). We applied RNA- and DNA-based reverse genetics methods to design and rescue four ICs of LGTV. The RNA-based method utilized in vitro transcribed LGTV genomic RNA as described by Lindqvist et al.^12^, whereas the DNA-based method utilized overlapping subgenomic amplicons as described by Aubry et al.^13^.

The discontinued use of live LGTV in earlier clinical trials (Gritsun et al.^7^), underscores the importance of further attenuation of the virus to enhance safety while maintaining immunogenicity. This provides the rationale for developing a reverse genetics LGTV platform to systematically introduce targeted mutations. The long-term goal of this study is to exploit the potential of reverse genetics to further attenuate LGTV and develop a live-attenuated TBE vaccine. Over the last few decades, reverse genetics has transformed studies of important aspects of virus biology and the development of live-attenuated vaccines through genetic manipulations. The technique has led to the development of vital vaccines, including the novel oral poliovirus vaccine and the influenza vaccine^14,15^. Moreover, two orthoflavivirus vaccines developed by reverse genetics are approved for human use, and several promising candidates are currently in preclinical and clinical trials^6,16,17^.

Despite its immense potential, reverse genetics has yet to be augmented to produce a live-attenuated TBE vaccine, since all commercially available TBE vaccines are based on inactivated TBEV particles^18^. In this study, we passaged the rescued LGTV ICs in cell culture and assessed their genetic integrity and stability by next generation sequencing (NGS). In addition, we performed in vitro and in vivo characterization of one of the rescued LGTV ICs and compared it with the LGTV strain in our laboratory. This study provides a crucial foundation for developing a more robust TBE vaccine capable of providing long-lasting immunity.

## Results

### Genetic difference between our laboratory LGTV and the virus reference at GenBank

Orthoflaviviruses are known to undergo genetic adaptation during passaging in mammalian cells. The LGTV strain TP21 in our laboratory (LGTV_Lab_) has an unknown passage history and we were interested to investigate the genetic changes occurred under cell culturing conditions. To do so, we sequenced the LGTV_Lab_ and compared it with the original TP21 sequence in GenBank (NC_003690.1). Our analysis revealed ten mutations within the open reading frame of the LGTV genome. Six of these mutations resulted in amino acid change, whereas the remaining four were silent mutations (Table 1).

**Table 1:** List of genetic differences between LGTV TP21 and LGTV_Lab_

Position (nt)	LGTV TP21	LGTV_Lab_	Gene	Amino acid change
188	A	G	C	Lys → Glu
970	G	T	prM	No
1338	A	G	E	Asp → Gly
2282	C	T	E	His → Tyr
6091	C	T	NS3	No
6766	G	A	NS4A	No
8451	C	G	NS5	Thr → Ser
9288	G	A	NS5	Arg → Lys
9734	G	A	NS5	Asp → Asn
9769	T	C	NS5	No

### Rescue of LGTV ICs

**Fig. 1:**
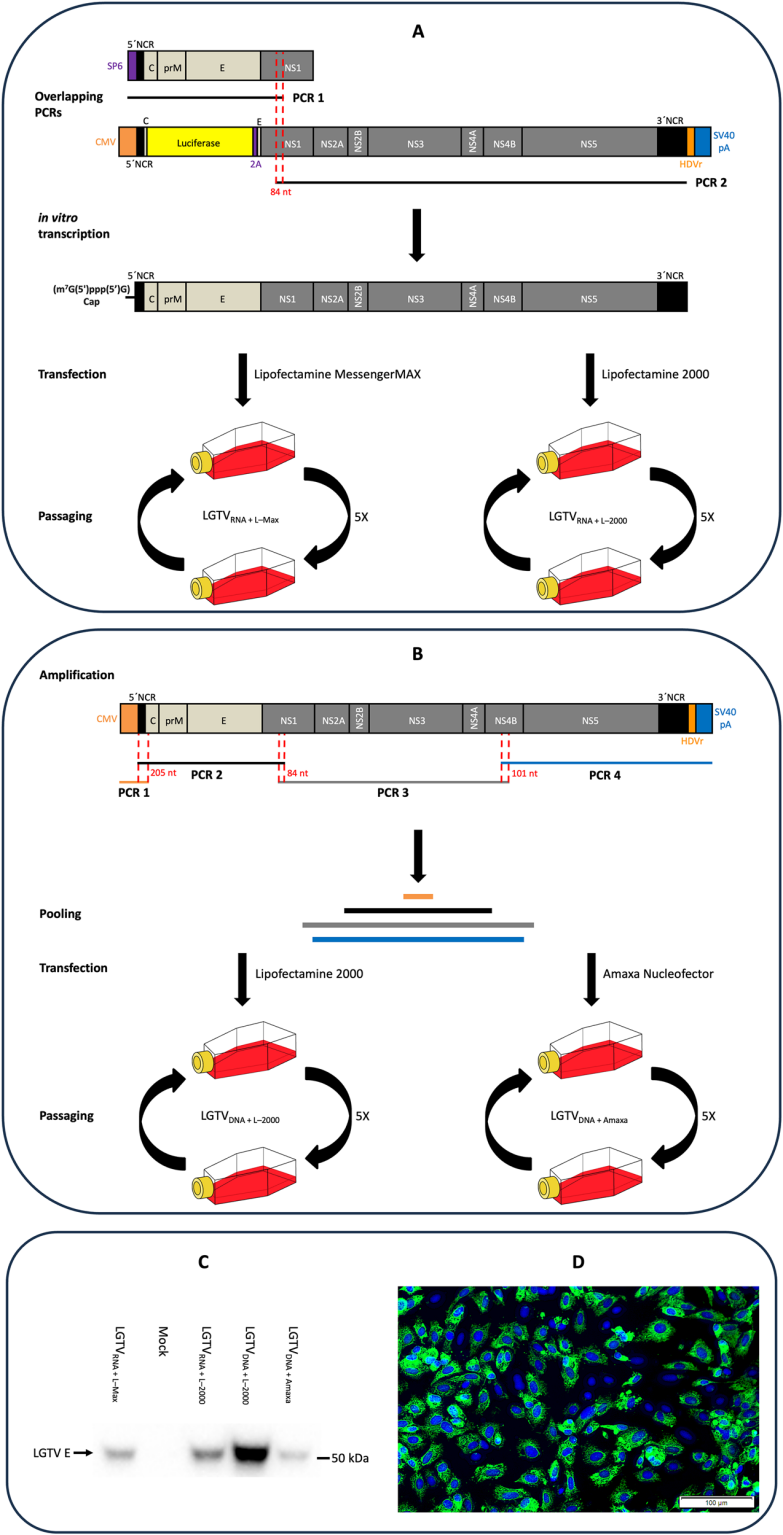
Rescue of LGTV infectious clones by reverse genetics. **A** Schematic illustration of RNA-based rescue of LGTV by amplification of two overlapping DNA fragments (fragment 1 and 2) to produce final DNA fragment 3 that comprises SP6 promoter and LGTV genome. The final DNA fragment was in vitro transcribed to produce LGTV RNA with a m^7^G(5´)ppp(5´)G cap before transfecting into cells. **B** Schematic illustration of DNA-based rescue of LGTV by four overlapping DNA fragments comprising cytomegalovirus promoter, LGTV genome, the hepatitis delta ribozyme and the simian virus 40 polyadenylation signal. The overlapping fragments were amplified by PCR and mixed in equimolar ratio before transfecting into cells. The viruses rescued by RNA- and DNA-based methods were passaged five times in cell culture. **C** Immunoblotting of cell culture media collected at day 7 from the transfected cells. Detection was performed using anti-LGTV E antibody. **D** Immunofluorescence assay of Vero E6 cells infected with LGTV_RNA+L–2000_. Staining was performed using anti-TBEV E primary antibody and Alexa 488 secondary antibody.

As part of our efforts to develop a live-attenuated TBE vaccine, we generated LGTV ICs. We successfully rescued four LGTV ICs based on TP21 sequence in GenBank (NC_003690.1): LGTV_RNA+L–Max_, LGTV_RNA+L–2000_, LGTV_DNA+L–2000_, and LGTV_DNA+Amaxa_, using RNA- and DNA-based reverse genetic methods and different transfection reagents, as detailed in Methods section and illustrated in Fig. 1. Rescue of LGTV ICs was confirmed by immunoblotting and immunofluorescence at 7 days post transfection (dpt). In addition, infectivity of these rescued viruses was confirmed by immunofluorescence assay at 3 days post infection (dpi). Notably, LGTV_DNA+L–2000_ showed better virus yield at 7 dpt compared to the other three ICs. The rescued viruses were subsequently passaged five times in cell culture.

### LGTV_DNA+L–Max_ and LGTV_DNA+L–2000_ showed higher genetic stability

**Fig. 2:**
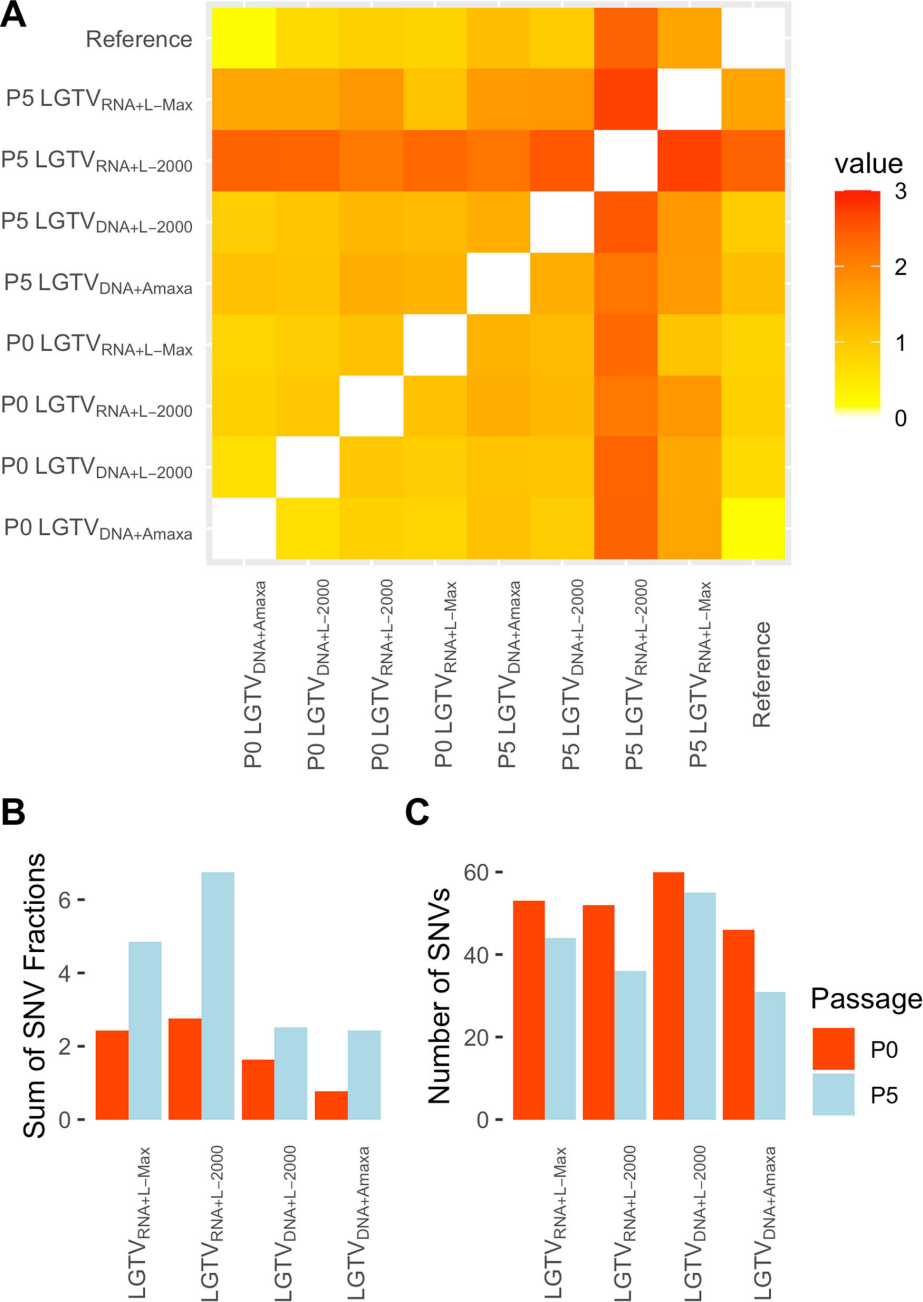
Genetic difference between the rescued infectious clones. **A** Heatmap based on the allele fractions to show the genetic difference between the different infectious clones as well as the reference LGTV sequence. The color is scaled according to the relative fraction of alternative alleles. The sum of single nucleotide variant (SNV) fractions (**B**) and the number of SNVs (**C**) of each infectious clone called with SNV fraction of 0.01 or higher.

LGTV ICs from passage-0 (p0) and passage-5 (p5) were sequenced by NGS to analyze their genetic stability during rescue and their adaptation under cell culture conditions. The ICs rescued by DNA-based method showed better stability as compared to the viruses rescued by RNA-based methods (Fig. 2). LGTV_DNA+L–Max_ and LGTV_DNA+L–2000_ were genetically more similar to the reference LGTV sequence as shown by the heat map and the sum of single nucleotide variant (SNV) fractions. The sum of SNV fractions was higher for all p5 ICs as compared to p0, which may reflect the accumulation of certain SNVs that confer advantage for growth in mammalian cells. Despite noticeable differences in the sum of SNV fractions, the total number of SNVs was quite similar between LGTV ICs rescued by both the DNA- and RNA-based methods. Interestingly, the number of SNVs was lower at p5 as compared to p0, possibly due to the selective pressure during cell culture adaptation.

### Passaging LGTV ICs in cell culture showed selection and emergence of SNVs

**Fig. 3:**
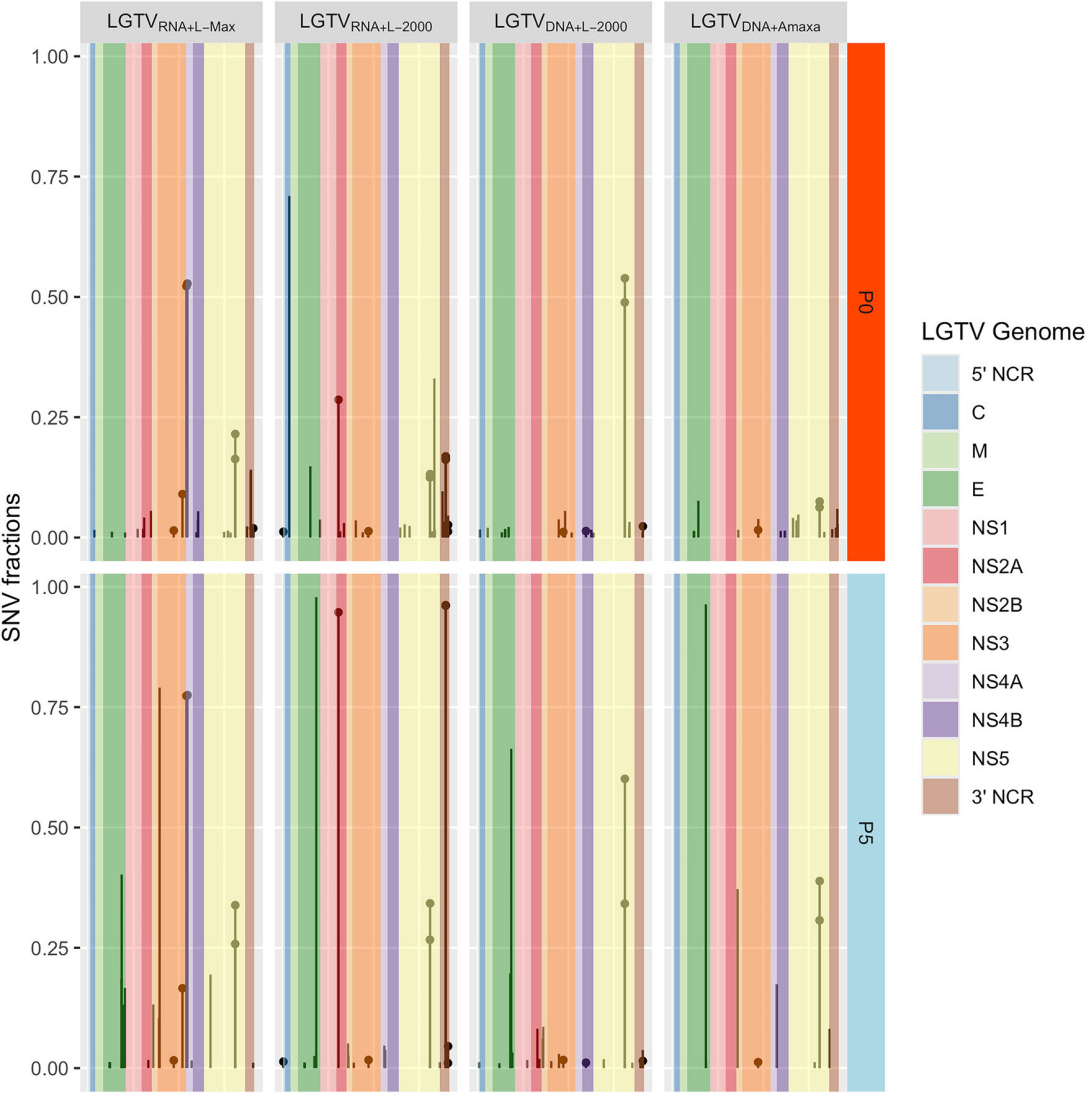
Distribution of single nucleotide variants (SNVs) within LGTV genome of the infectious clones. SNVs present in both passage 0 and 5 are highlighted by round dots.

Orthoflaviviruses are known to undergo selection pressure during cultivation in the laboratory. NGS analysis of the rescued viruses from p0 and p5 revealed the presence of LGTV quasispecies. We further analyzed the NGS data to characterize the distribution of SNVs across the LGTV genome. The SNVs were distributed over the whole LGTV genome. In general, the SNVs fractions were higher in ICs at p5 as compared to p0 which could be due to the preferential selection of those SNVs during cultivation in Vero E6 cells. Passaging the LGTV ICs in cell culture showed amplification of some SNVs already present at p0 as well as emergence of new SNVs (Fig. 3 and Supplementary Table 1). The SNVs detected in LGTV ICs at position 2282 (H718Y) and 9734 (D3202N) were also identified in LGTV_Lab_, as mentioned earlier (Table 1). Interestingly, these SNVs were present at much lower levels in all the rescued LGTV ICs at p0 as compared to p5, and their levels increased during cultivation in Vero cells (Supplementary Table 1).

### LGTV_DNA+L–2000_ exhibited an in vitro profile comparable to LGTV_Lab_

**Fig. 4:**
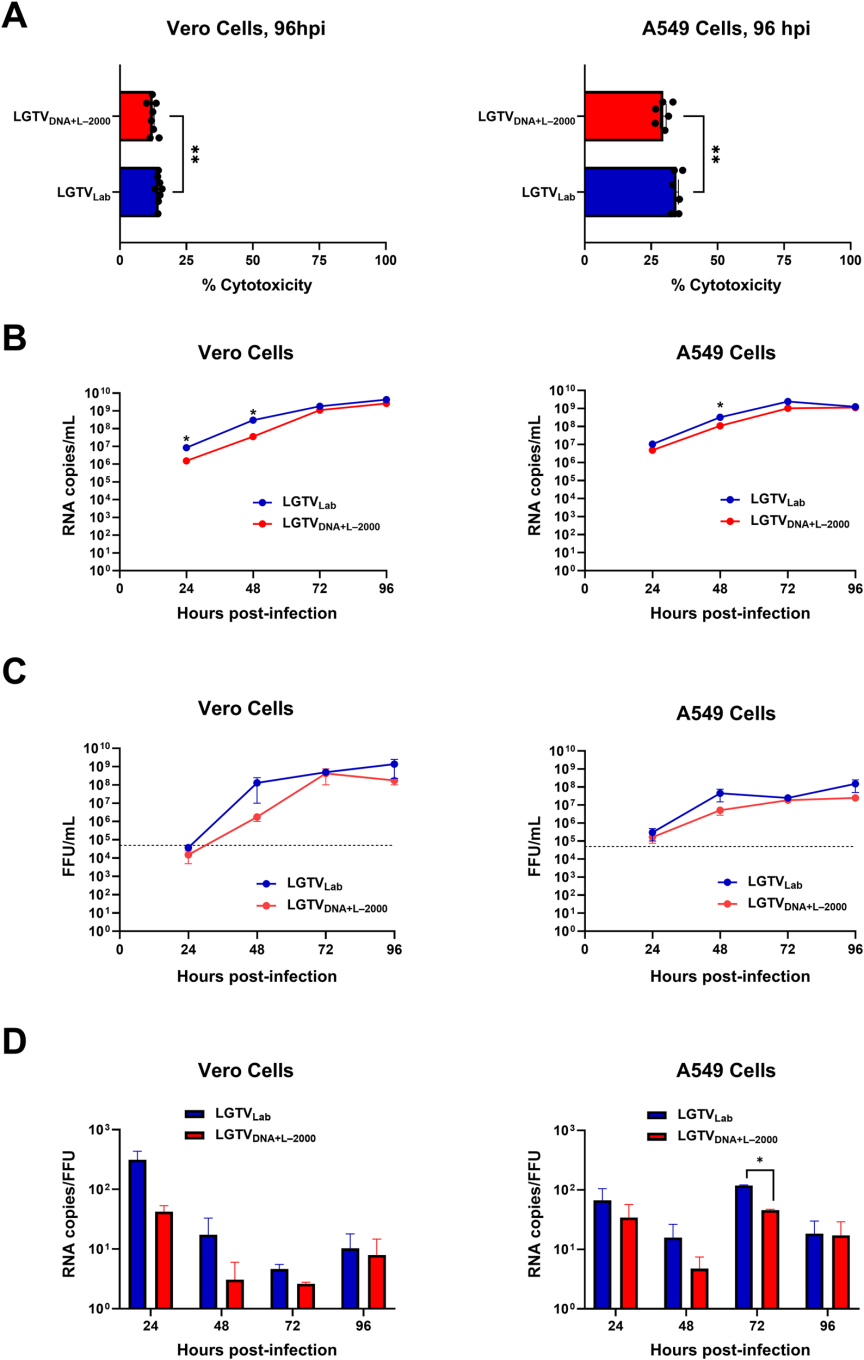
In vitro characterization of LGTV_DNA+L–2000_ from passage 6 and LGTV_Lab_ in Vero and A549 cells. **A** Percent cytotoxicity determined by lactate dehydrogenase (LDH) cytotoxicity assay at 96 hpi. The experiments were conducted in octuplicate (Vero) or sextuplicate (A549), and absorbance was measured in technical triplicates. **B** LGTV growth kinetics represented as RNA copies per mL of cell culture media. The experiments were run in triplicates. **C** LGTV growth kinetics represented as infectious virus titers at indicated time points. The experiments were run in duplicates. **D** LGTV infectivity represented as RNA copies to FFU ratios at indicated time points. Specific infectivities were calculated using the same samples and time points, run in duplicates. The error bars represent SEM, and significant differences are indicated (*p < 0.05, **p < 0.01).

As LGTV_DNA+L–2000_ showed better virus yield and was genetically closer to the reference LGTV sequence, we decided to characterize it further. We evaluated the cytotoxicity, replication and infectivity profiles of the rescued IC LGTV_DNA+L–2000_ and LGTV_Lab_ in Vero E6 and A549 cells using a multiplicity of infection (MOI) of 0.05 (Fig. 4). Cytotoxicity induced by LGTV_DNA+L–2000_ was significantly lower (*p* < 0.01) than that of LGTV_Lab_, in both Vero and A549 cells. Both LGTV_DNA+L–2000_ and LGTV_Lab_ showed progressive increases in LGTV RNA levels in cell culture, indicating active replication. However, LGTV_DNA+L–2000_ exhibited lower RNA levels than LGTV_Lab_, with significant differences (*p* < 0.05) at 24h post infection (hpi) in Vero cells and at 48 hpi in both cell types. Peak levels of LGTV RNA varied by cell type, occurring at 72 hpi in A549 cells and 96 hpi in Vero cells. LGTV_DNA+L–2000_ and LGTV_Lab_ showed similar infectious titers that increased over time. In A549 cells, peak infectious titers for both strains were observed at 96 hpi, while peak titers in Vero cells were observed at 72 hpi for LGTV_DNA+L–2000_ and at 96 hpi for LGTV_Lab_. Although the infectious titers of LGTV_DNA+L–2000_ were consistently lower than LGTV_Lab_, the differences were not statistically significant. LGTV_DNA+L–2000_ showed a lower RNA copies/focus-forming units (FFU) ratio as compared to LGTV_Lab_ which indicated higher specific infectivity of LGTV_DNA+L–2000_. A549 cells exhibited comparatively higher levels of defective virus particles as compared to Vero cells. In summary, both strains exhibited a similar in vitro profile, but LGTV_DNA+L–2000_ showed slightly lower genomic RNA and infectious titers but higher infectivity compared to LGTV_Lab_, suggesting a relatively attenuated replication that in turn contributed to less defects in virion assembly.

### LGTV_DNA+L–2000_ and LGTV_Lab_ exhibited similar in vivo profiles

**Fig. 5:**
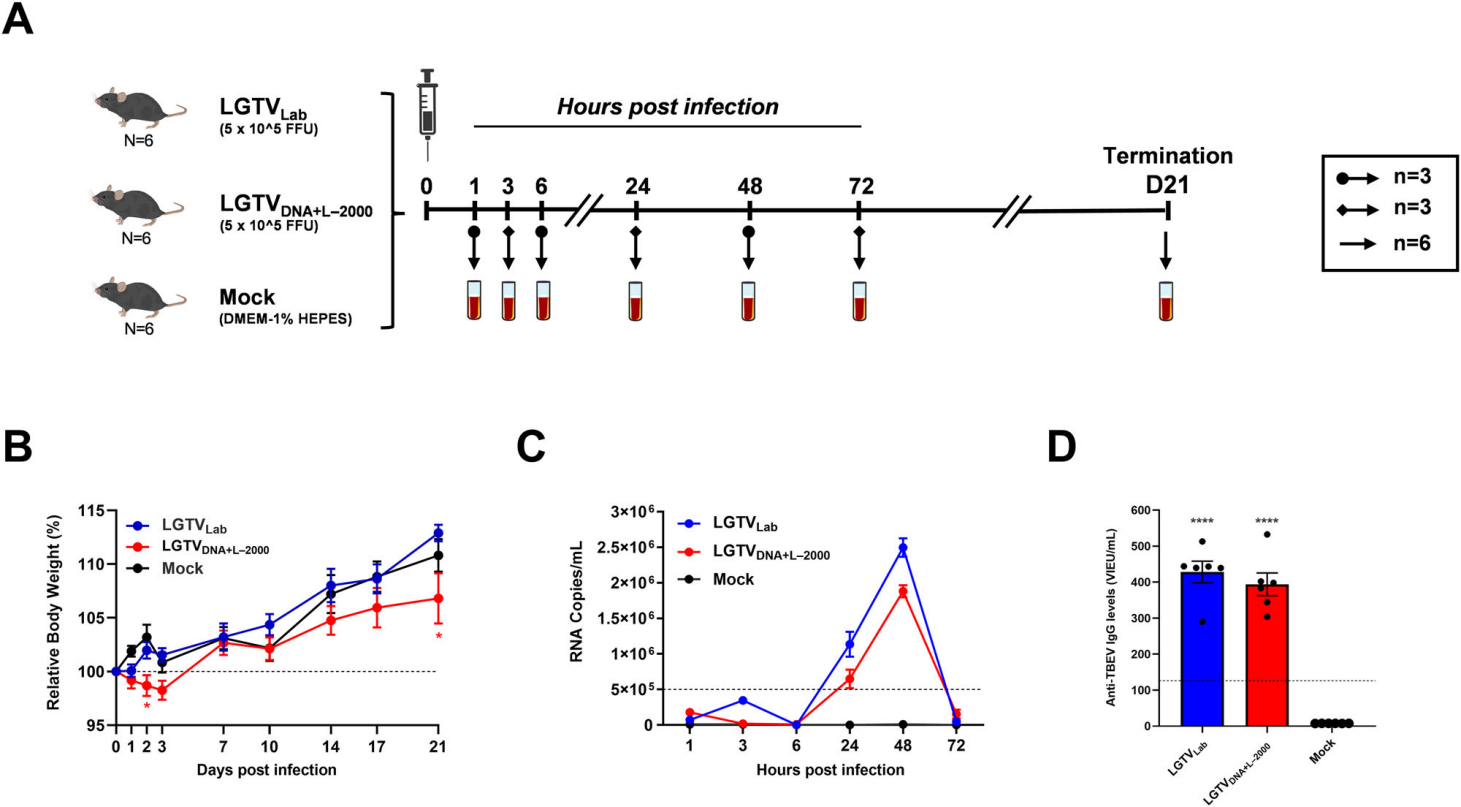
In vivo evaluation of LGTV_DNA+L–2000_ from passage 6 and LGTV_Lab_ in C57BL/6J mice. **A** Schematic illustration of study layout showing mice groups, infection plan and sampling time points. Mice sera from all animals (*n* = 6) or half of the animals (*n* = 3) in each group were collected at different time points. **B** Relative body weights measured at different time points and presented as percentage of initial body weight of each mouse. **C** Viremia presented as mean RNA copies per mL at indicated time points. **D** Levels of anti-TBEV IgG antibodies in mice sera at 21 dpi. Bars represent the standard deviations of mean IgG levels per group. A threshold for positive samples was set to 126 Vienna Units per mL (VIEU/mL) as per manufacturer’s instructions. Significance was calculated in comparison to the control mice (*p < 0.05, ****p < 0.001), and error bars represent SEM.

In vivo evaluation of LGTV_DNA+L–2000_ and LGTV_Lab_ was performed in C57BL/6 J mice. Mice infected with 5 × 10^5^ FFU of LGTV_DNA+L–2000_ or LGTV_Lab_ by intramuscular (IM) injection were monitored for general health status and body weight for any potential side effects (Fig. 5). Mice infected with LGTV_DNA+L–2000_ exhibited an initial decrease in body weight between days 1 and 3, followed by recovery and progressive increase in body weight. The relative body weights of LGTV_DNA+L–2000_ group were significantly lower (*p* < 0.05) on days 2 and 21, compared to both, the control mice and those infected with LGTV_Lab_ (Fig. 5B). No notable changes were observed in the general health status of mice infected with LGTV_Lab_ or the control mice. Most of the mice infected with LGTV_DNA+L–2000_ remained asymptomatic, while one mouse developed a wound at the injection site (Supplementary Note 1). The wound initially showed signs of healing by day 7 but worsened by the end of the study. While this localized reaction occurred in only one mouse, it suggests the potential of injection-site irritation associated with intramuscular administration of LGTV_DNA+L–2000_.

Viremia in both LGTV_DNA+L–2000_ and LGTV_Lab_ infected mice exhibited similar profiles, with peaks observed at approximately 48 hpi (Fig. 5C). However, viremia was detected earlier (1 hpi) in LGTV_DNA+L–2000_ group compared to LGTV_Lab_ (3 hpi). Following an initial decline at 6 hpi, viremia levels for both strains increased at 24 hpi and reached the peak at 48 hpi. Notably, LGTV_DNA+L–2000_ exhibited lower viral loads than LGTV_Lab_. In addition, we measured the humoral immune response by quantifying TBEV cross-reactive antibodies in sera collected at 21 dpi. The anti-TBEV IgG levels in both LGTV_DNA+L–2000_ and LGTV_Lab_ mice groups were significantly higher (*p* < 0.0001) than those of the control mice (Fig. 5D). There was no significant difference in anti-TBEV IgG levels between LGTV_DNA+L–2000_ and LGTV_Lab_ groups. In summary, these findings indicated that LGTV_DNA+L–2000_ was tolerated by C57BL/6J mice, it could replicate and disseminate to animal blood, and it elicited humoral immune response comparable to that of LGTV_Lab_.

## Discussion

Over the past few decades, reverse genetics has significantly advanced our understanding of biology and replication cycle of single stranded RNA viruses. Most reverse genetics systems require construction of infectious cDNA plasmids that encode manipulated viral genomes. We attempted to rescue LGTV ICs by constructing an infectious cDNA plasmid, but we were unable to produce a stable plasmid for amplification in *Escherichia coli* (data not shown). Plasmids containing orthoflavivirus cDNAs have previously been shown to be highly unstable^19^. In addition, the presence of cryptic prokaryotic promoter sequences within the orthoflavivirus genome may generate peptides that are toxic to *E. coli*, which could further complicate the process^20^. To overcome these problems, we used reverse genetics approaches described by Aubry et al.^13^, and Lindqvist et al.^12^.

In this study, we utilized RNA- and DNA-based reverse genetic strategies to rescue four infectious clones of LGTV. The RNA-based method utilised full length LGTV RNA transcripts that were synthesized and capped in vitro before cellular transfection. The transfected RNA transcripts harnessed the cellular translational machinery to initiate orthoflavivirus replication cycle to produce LGTV_RNA+L–Max_ and LGTV_RNA+L–2000_ virions. We have previously used Lipofectamine 2000 for rescue of TBEV ICs by RNA-based reverse genetics^12,21^. In this study, we used Lipofectamine MessengerMAX in parallel to Lipofectamine 2000 for RNA transfection, because the former is claimed to provide two-fold higher transfection efficiency as compared to Lipofectamine 2000. The DNA-based method required transfection of four overlapping DNA fragments to exploit the homologous recombination within mammalian cells. There was an overlap of 205 bp between amplicon 1 and 2, an overlap of 84 bp between amplicon 2 and 3, and an overlap of 101 bp between amplicon 3 and 4 (Fig. 1B), which facilitated homologous DNA recombination to generate full length DNA fragments comprising human cytomegalovirus (CMV) promoter, complete LGTV genome, the hepatitis delta ribozyme (HDVr) and the simian virus 40 polyadenylation signal (SV40 pA). The CMV promoter facilitated transcription of LGTV RNA by cellular DNA dependent RNA polymerase, whereas SV40 pA and HDVr facilitated transport of LGTV RNA transcripts from nucleus into cytoplasm and post-transcriptional processing to initiate the virus replication cycle to produce LGTV_DNA+L–2000_ and LGTV_DNA+Amaxa_ virions. A co-culture of HEK-293 and Vero E6 cells was used to rescue LGTV ICs where the former facilitates transfection, and the latter is more compatible for virus propagation. Moreover, we applied the Nucleofector program Q-001 (Lonza, Basel, Switzerland) to transfect the co-culture of Vero E6 and HEK-293 cells. This program is optimized for HEK-293 cells and not for Vero cells. We used Q-001 to benefit from the transfection potential of HEK-293.

TBEV exists as a pool of variants called quasispecies which undergo adaptation in response to selection pressure during propagation in mammalian cells^21,22^. NGS analysis of the rescued viruses confirmed the presence of quasispecies within LGTV ICs. It has been shown that viable TBEV variants can exist at abundances of less than 1% in the long-term and can become a major part of the viral population under favorable conditions^23^. In this study, several SNVs with an abundance of ∼1% were identified in the E protein of all LGTV ICs from p0 and p5 (Supplementary Table 1). The orthoflavivirus E protein is required for cellular infection through receptor mediated endocytosis and the diversity observed in the E protein of LGTV quasispecies may facilitate LGTV ICs to infect a broader range of cells. Passaging LGTV ICs in cell culture led to both the selection of existing variants and the emergence of new variants. Sanger sequencing of LGTV_Lab_ revealed ten SNVs compared to the original TP21 sequence in GenBank. Interestingly, two of these ten SNVs, H718Y and D3202N, were also detected in all the rescued LGTV ICs and their levels increased during subsequent propagation in Vero cells. These observations suggest that H718Y and D3202N are clearly an in vitro phenomenon, occurring primarily in cell culture. Interestingly, the rescued viruses from p0 showed comparatively higher number of SNVs as compared to those from p5, suggesting that the viruses experienced strong selection pressure during initial culturing. Conversely, the rescued viruses from p5 showed a higher sum of SNV fractions as compared to p0, reflecting virus adaptation over time while cultivating in mammalian cells. Similar observations of selecting existing variants as well as emergence of new variants under cell culturing conditions have also been reported for TBEV^21,22^.

In vitro characterization of LGTV_DNA+L–2000_ and LGTV_Lab_ showed that the peak levels of LGTV RNA reached quicker in A549 cells as compared to Vero cells. In contrary, Vero cells exhibited lower cytotoxicity and higher virus titers as compared to A549 cells. In addition, production of defective virus particles was lower in Vero cells as compared to A549 cells. These differences in growth kinetics and specific infectivity could be related to the differences in the immune profiles of these cell lines. A549 cells are known to produce interferon whereas Vero cells are deficient in type I interferon production, which makes Vero cells more compatible for virus cultivation^24,25^. The comparison of in vitro growth kinetics of both LGTV strains revealed that both the genomic RNA and infectious titers of LGTV_DNA+L–2000_ were slightly lower than those of LGTV_Lab_. Notably, LGTV_DNA+L–2000_ also exhibited significantly lower in vitro cytotoxicity. The combination of reduced viral replication and lower cytotoxicity is particularly appealing for development of a live-attenuated TBE vaccine, as replication-defective viruses have effectively been used as live-attenuated viral vaccines^26^.

In a murine model, LGTV_DNA+L–2000_ infection was associated with an initial decline in body weight followed by recovery and progressive increase over time, but it was significantly lower than LGTV_Lab_ and control mice, at the termination of the experiment. The difference in the weight development of mice infected with LGTV_DNA+L–2000_ and LGTV_Lab_ could be due to genetic differences between the two viruses. In addition, the initial decline in body weight may be due to the activation of the early immune responses, including cytokine release and inflammatory responses, which are essential for protection against infection^27^. A steadier increase in body weight of mice in LGTV_Lab_ group as compared to those in LGTV_DNA+L–2000_ group, reflects better tolerance of the former. This more attenuated phenotype of LGTV_Lab_ as compared to LGTV_DNA+L–2000_ could be attributed to the passage history of the virus because serial passaging is a well-documented method for virus attenuation, particularly in the development of live-attenuated vaccines^26,28–30^. Following rescue, LGTV_DNA+L–2000_ was passaged only five times in cell culture whereas LGTV_Lab_ has been cultured in laboratory for several decades. Additionally, the localized irritation observed in one mouse in this study is the most common reaction to intramuscular administration, which is generally not serious and fades away by itself^31^. This reaction could be avoided by using another administration route such as mucosal administration, which requires further research.

To our knowledge, this is the first study demonstrating the viremia kinetics of a laboratory adapted and a rescued IC of LGTV TP21 strain in a murine model. To study viremia kinetics, blood samples were collected at 1, 6 and 48 h from 50% of the mice and at 3, 24, and 72 h from the remaining 50% animals in each group. Blood sampling from different mice may have introduced variability in viremia assessment. This was done according to the ethical guidelines where a maximum blood volume of 8 μL/g is allowed to be sampled over a period of 14 days. This equates to approximately 53 μL/time-point for the in-life samples, provided that the animals weigh 20 g. A recent study by Ahmed et al.^32^ investigated LGTV acquisition dynamics in female C57BL/6 mice by intraperitoneal inoculation with 4 × 10^5^ FFU of LGTV. Viremia was detected at 3 dpi and 6 dpi, indicating a higher virus load at 3 dpi^32^. In contrast, our data demonstrated that both LGTV_DNA+L–2000_ and LGTV_Lab_ reached peak viremia at 2 dpi and the virus was subsequently cleared by 3 dpi. The differences between our findings and those of Ahmed et al.^32^ may be attributed to the different viral doses and administration routes used in the two studies. Notably, LGTV_DNA+L–2000_ exhibited a lower viremia level compared to LGTV_Lab_. These findings advocate further investigation of LGTV_DNA+L–2000_ as a potential vaccine candidate, because it exhibited efficient replication and rapid viral clearance that may minimize adverse effects in vivo.

Live-attenuated vaccines have been successful in protecting from yellow fever, Dengue fever and Japanese encephalitis^16,33–35^. It has recently been shown that LGTV infection in a murine model produced both cross-reactive antibodies and T cells against TBEV^36^. In addition, LGTV infection of C57BL/6J has shown to protect mice against TBEV challenge^37^. The ELISA kit used in this study is coated with inactivated central European TBEV. The kit was selected due to similar antigenic profile of TBEV and LGTV, and production of cross-reactive antibodies^10,36^. In this study, we selected LGTV_DNA+L–2000_ for further in vitro and in vivo characterization because DNA-based methods offer higher stability compared to RNA-based methods, and it saves time and resources to generate additional LGTV ICs for the purpose of developing a live-attenuated TBE vaccine. The level of anti-TBEV IgG antibodies produced by LGTV_DNA+L–2000_ was comparable to that of LGTV_Lab_. The induction of immune response by LGTV_DNA+L–2000_ to produce cross-reactive anti-TBEV antibodies highlight its potential to develop a live-attenuated TBE vaccine. We used a single dose to evaluate immunogenicity in mice which may limit the interpretation of the immunogenicity of our vaccine candidate. However, the clinical trials of live LGTV conducted in 1970s, showed that a single dose of live vaccine produced long-lasting seroconversion in 100% of individuals, as reviewed by Gritsun et al.^7^.

In conclusion, LGTV_DNA+L–2000_ that was rescued by DNA-based reverse genetics method showed higher genetic stability during rescue and cell culturing, it closely resembled LGTV_Lab_ in terms of in vitro and in vivo replication kinetics, and it induced a humoral immune response in mice which was comparable to LGTV_Lab_. These findings provide valuable insights into the potential use of LGTV_DNA+L–2000_ as a platform for vaccine development. However, further attenuation and more thorough evaluation of the cellular immune response are necessary for its use as a live-attenuated TBE vaccine. Virus-specific humoral and cellular immunity is required to provide robust protection against TBEV^4^. Regarding the cellular immune response, CD4 + T-cells have shown to play a protective role by restricting the infection while CD8 + T-cells may contribute to the severity of TBE^38^. Despite certain limitations, our findings present LGTV_DNA+L–2000_ as a promising platform for TBE vaccine development. The methods described for generating and rescuing LGTV_DNA+L–2000_ enable the introduction of targeted mutations for further attenuation. Such mutation may target key residues within LGTV NS3 and NS5 proteins as well as 3´NCR which are important for virus replication and virus-host protein interaction. The ongoing research in our group is focused on developing attenuated variants of LGTV_DNA+L–2000_ using reverse genetics, which can be used as a live-attenuated TBE vaccine.

## Methods

### Cells

Human embryonic kidney cells HEK-293 (CRL-1573_ ATCC), African green monkey kidney cells Vero E6 (CRL-1586_ ATCC) and human lung epithelial cells A549 (CCL-185_ ATCC), were maintained in Dulbecco’s Modified Eagle’s Medium (DMEM) containing 1 g/L glucose (Gibco, Thermo Fisher Scientific, Waltham, MA, USA), supplemented with 10% heat-inactivated fetal bovine serum (HI‒FBS, Gibco, Thermo Fisher Scientific, Waltham, MA, USA) and 100 units/ml penicillin-streptomycin (Gibco, Thermo Fisher Scientific, Waltham, MA, USA). The cells were maintained at 37 °C under 5% CO_2_.

### Viruses

The LGTV strain TP21 was isolated in 1956 from *Ixodes granulatus* tick near Kuala Lumpur, Malaysia^39^. TP21 has been propagated in laboratory for several decades. The TP21 strain in our laboratory (hereafter referred to as LGTV_Lab_) was a kind gift from Prof. Anna K. Överby (Department of Clinical Microbiology, Virology, Umeå University, Sweden). LGTV_Lab_ has been propagated in Vero B4 and Vero E6 cells, but the exact passage history is unknown. We rescued four LGTV ICs based on LGTV strain TP21 sequence in GenBank (NC_003690.1) using RNA- and DNA-based methods. The ICs were named LGTV_RNA+L–Max_, LGTV_RNA+L–2000_, LGTV_DNA+L–2000_ and LGTV_DNA+Amaxa_, based on the strategy and the transfection reagent used.

For RNA-based rescue, two overlapping DNA fragments (fragment 1 and 2) were amplified and used as template for an overlapping PCR to generate the full-length fragment 3 using the primers listed in Table 2. Fragment 3 contained the SP6 promoter and the complete LGTV genome. Fragment 3 was used as template for in vitro transcription to synthesize capped LGTV RNA using the MEGAscript® SP6 Kit, and the cap analog m^7^G(5´)ppp(5´)G from Invitrogen (Thermo Fisher Scientific, Waltham, MA, USA) as per the manufacturer’s instructions. Lipofectamine MessengerMAX and Lipofectamine 2000 from Invitrogen (Thermo Fisher Scientific, Waltham, MA, USA) were used for transfecting the in vitro transcribed RNA to rescue LGTV_RNA+L–Max_ and LGTV_RNA+L–2000_ viruses, respectively.

**Table 2:** List of primers used to amplify different fragments for the rescue of LGTV infectious clones

		Primer name	Primer sequence
Overlapping PCR for RNA-based rescue	Fragment 1	SP6 forward	ATCGATTTAGGTGACACTATAGAG
		NS1 reverse	GAAGACCAATCCCAAACTCT
	Fragment 2	NS1 forward	TTGGAGTGGAAGGAGGTAGA
		3NCR reverse	AGCGGGTGTTTTTCCGA
	Fragment 3	SP6 forward	ATCGATTTAGGTGACACTATAGAG
		3NCR reverse	AGCGGGTGTTTTTCCGA
Overlapping amplicons for DNA-based rescue	Amplicon 1	CMV forward	CATGTTTGACAGCTTATCATCG
		Capsid reverse	CTTCTTTGGGGCCACTTTC
	Amplicon 2	5NCR forward	AGATTTTCTTGCGCGTG
		NS1 reverse	GAAGACCAATCCCAAACTCT
	Amplicon 3	NS1 forward	TTGGAGTGGAAGGAGGTAGA
		NS4B reverse	AGAAGAACACTTTGTGAGCC
	Amplicon 4	NS4B forward	ATCTTTAATCCTGGGAGTCG
		SV40 reverse	TCGGTCGATGCTCTAGATAC

For the DNA-based method, four overlapping DNA amplicons were produced using the primers listed in Table 2. The first amplicon comprises the human CMV promoter and LGTV 5´NCR-C. The second and third amplicons contain 5´NCR-NS1 and NS1-NS4B sequence of LGTV, respectively. The fourth amplicon comprises NS4B-3´NCR of LGTV, HDVr and SV40 pA. PCR products were analyzed by gel electrophoresis and gel purified by Wizard® SV Gel and PCR Clean-Up System (Promega, Madison, WI, USA). The four amplicons were mixed in equimolar ratio before transfection into cells using Lipofectamine 2000 (Invitrogen, Thermo Fisher Scientific, Waltham, MA, USA) or Amaxa nucleofector (Lonza, Basel, Switzerland) to rescue LGTV_DNA+L–2000_ and LGTV_DNA+Amaxa_ viruses, respectively.

### Cell transfection

For transfection with Lipofectamine 2000 or Lipofectamine MessengerMAX, a mixture of HEK-293 and Vero E6 cells (1:1) was seeded in a 6 well plate (600,000 cells/well). The in vitro transcribed RNA (500 ng) or equimolar DNA amplicons (600 ng) were transfected using OptiMEM medium (Gibco, Thermo Fisher Scientific, Waltham, MA, USA) as per manufacturer’s instructions. After 4 h of incubation, transfection medium was replaced with DMEM medium supplemented with 2% fetal bovine serum and 100 U/mL penicillin-streptomycin (all from Gibco, Thermo Fisher Scientific, Waltham, MA, USA). For transfection with Amaxa nucleofector (Lonza, Basel, Switzerland), 10^6^ HEK-293 and Vero E6 cells (1:1) were electroporated with 600 ng of equimolar amplicon-mix using Nucleofector program Q-001. After transfection, 600,000 and 400,000 cells were seeded into two separate wells of a 6-well plate. As a negative control, 1 million cells were electroporated with PBS and 400,000 cells were seeded into a separate well.

### Sequencing of the LGTV genome

LGTV genome was amplified into eigth overlapping DNA fragments using the primers listed in Table 3 and KOD Hot Start Master Mix (Novagen, Merck, Darmstadt, Germany). The PCR products were gel purified using the Wizard® SV Gel and PCR Clean-Up System (Promega, Madison, WI, USA) before sequencing. LGTV_Lab_ was sequenced using Sanger sequencing (Eurofins Genomics, Ebersbeg, Germany) whereas LGTV ICs were sequenced by NGS. For NGS, the overlapping DNA fragments were pooled in a 1:1 molar ratio. The Nextera® XT DNA library preparation kit (Illumina, San Diego, CA, USA) was used to prepare an indexed paired end multiplexed sequencing library as described previously^21^. Libraries were pooled after normalization and denatured with 0.2 N NaOH before loading onto a MiSeq cartridge v2, 300 cycles (Illumina, San Diego, CA, USA). NGS was performed with a MiSeq desktop sequencer (Illumina, San Diego, CA, USA) using index reads = 2 and paired-end settings.

**Table 3:** List of primers used to amplify eight overlapping fragments to sequence complete LGTV genome

Primer	Sequence	Position (nt)	PCR target
TP21 forward 1	AGATTTTCTTGCGCGTG	1-17	
TP21 reverse 1	CTGATGACACTGTGAACGA	1460-1479	5´NCR-E
TP21 forward 2	TATACCATAAAGGTGGAGCC	1385-1404	
TP21 reverse 2	GAAGACCAATCCCAAACTCT	2929-2948	E-NS1
TP21 forward 3	TTGGAGTGGAAGGAGGTAGA	2865-2884	
TP21 reverse 3	TTCTGGATTCCACTCAATGTT	4373-4393	NS1-NS2B
TP21 forward 4	GATGGGCATTCCAGTGAAG	3865-3883	
TP21 reverse 4	TCTCTGATATGTCCGTGGTC	5833-5852	NS2B-NS3
TP21 forward 5	AGTGTCATTTGCTTAAACAGC	5756-5776	
TP21 reverse 5	AGAAGAACACTTTGTGAGCC	7312-7331	NS3-NS4B
TP21 forward 6	ATCTTTAATCCTGGGAGTCG	7231-7250	
TP21 reverse 6	ATGCAGTTGTGTCAGTCATT	8692-8711	NS4B-NS5
TP21 forward 7	TCGTGAAGCTTCTCAGTTGG	8637-8656	
TP21 reverse 7	TCTTCCATGAATGGGTTGTC	10124-10143	NS5
TP21 forward 8	CAGGTTGTGACTTATGCTCT	9476-9495	
TP21 reverse 8	AGCGGGTGTTTTTCCGA	10927-10943	NS5-3´NCR

### NGS data analysis

The quality of the NGS data was assessed by generating quality-control statistics with Trimmomatic version 0.39^40^ using default parameters to remove Nextera adapters and additional parameters to only keep high quality reads (LEADING:3 TRAILING:3 MINLEN:36 SLIDINGWINDOW:30:20). Trimmed reads were aligned to the LGTV genome (NC_003690.1) using bwa mem^41^. The average sequencing depth per base across all samples was in the span 3473-4198x. SNVs were called using LoFreq^42^ and amino acid changes annotated with vcf-annotator (https://github.com/rpetit3/vcf-annotator) using data from RefSeq (BioProject: PRJNA485481). For analyses of SNVs across multiple samples, all bases were inspected using a custom script^43^ and positions with allele-fraction ≥ 0.01 were used for analysis. All subsequent analyses and visualizations were done in Rstudio using R version 4.0.2.

### Virus cultivation and stock

The rescued viruses were passaged five times in Vero E6 cells. Virus stocks of LGTV_DNA+L–2000_ and LGTV_Lab_ were prepared for in vitro and in vivo characterization. Briefly, Vero E6 cells were infected with LGTV_DNA+L–2000_ from passage 5 or LGTV_Lab_ at a MOI of 0.05. Media from the infected cells were collected 5 dpi and centrifuged at 4000 × *g* for 10 min to remove dead cells. The clarified supernatants were layered onto a 25% sucrose cushion, and virus particles were pelleted by ultracentrifugation at 150,000 × *g* for 2 h at 4 °C. The resulting virus pellets were gently resuspended in small volumes of DMEM containing 1% HEPES (Invitrogen, Thermo Fisher Scientific, Waltham, MA, USA), pooled, aliquoted, and stored at −80 °C for future use. The infectious virus titer was determined using a focus forming assay (FFA) as previously described^44^, and the infectious virus concentration was calculated in FFU/mL.

### LGTV growth kinetics

The growth kinetics of LGTV_DNA+L–2000_ and LGTV_Lab_ were assessed using a similar protocol as previously described by Hoornweg et al.^45^ for TBEV. Briefly, monolayers of A549 or Vero E6 cells, seeded in 24-well plates, were inoculated with LGTV_DNA+L–2000_ from passage 6 or LGTV_Lab_ using MOI of 0.05. The inoculum was replaced with 2% HI–FBS medium after 2 h to remove the unbound virus particles, and the cells were further incubated at 37 °C. Media from the infected cells were collected at 24-h intervals until 4 dpi. LGTV RNA was quantified using real-time PCR (RT-qPCR), and infectious virus titers were determined via FFA. The experiments were performed in triplicates.

### Cytotoxicity assay

Cellular cytotoxicity following infection with LGTV_DNA+L–2000_ or LGTV_Lab_ was evaluated by measuring LDH release using the CyQUANT™ LDH Cytotoxicity Assay Kit (Thermo Fisher Scientific, Waltham, MA, USA), according to the manufacturer’s instructions. Briefly, A549 or Vero E6 cells were seeded in 96-well plates and infected with LGTV_DNA+L–2000_ from passage 6 or LGTV_Lab_ using 0.05 MOI. Cell culture medium was collected at 96 hpi to measure LDH activity. Maximum LDH release was determined by treating cells with lysis buffer for 30 min, while spontaneous LDH release was measured in medium from uninfected control cells collected at the same time. Percentage cytotoxicity was calculated according to the manufacturer’s instructions. The experiments were performed in sextuplicate (A549) or octuplicate (Vero), and absorbance was measured in technical triplicates.

### Mice immunization

Eight-week-old female C57BL/6J mice (*n* = 18) were housed at Scantox Sweden, Solna, Sweden.

The housing of the animals was in accordance with EU Directive 2010/63/EU of 22 September 2010 on the protection of animals used for scientific purposes. All animal experimentation was carried out under the animal ethical permit number 767-22, approved by The Swedish Board of Agriculture and the regional animal ethics committee in Stockholm. Mice (*n* = 6) in each group were infected with 5 × 10^5^ FFU of LGTV_DNA+L–2000_ from passage 6 or LGTV_Lab_ by IM injection in 50 µL DMEM containing 1% HEPES. Mice in the control group received an injection of 50 µL DMEM with 1% HEPES. IM administration was performed under 3.5% isoflurane anesthesia to minimize stress and pain. Needles were changed between animals to avoid unnecessary pain from blunt needles.

### Clinical observation and sampling from mice

The general health status of mice was monitored by recording body weight and clinical symptom scores. Body weight measurements were conducted weekly for a period of three weeks post-administration. Clinical assessments were performed using a standardized symptom scoring system. In-life blood samples were collected from puncture of the saphenous vein on conscious animals at 1, 3, 6, 24, 48, 72 hpi and from the orbital plexus of anaesthetized mice (ketamine/xylazine (90 mg/kg + 10 mg/kg i.p.)) on the termination day (D21). However, not all animals were sampled at each time point (except D21). Blood was sampled at 1, 6, and 48 h from half of the animals (*n* = 3) in each group and at 3, 24, and 72 h from the remaining three animals. Serum was immediately separated from blood by centrifugation at 10,000 × *g* for 5 min at room temperature and stored frozen at −80 °C until further analysis. Animals were euthanised by cervical dislocation following collection of the terminal blood sample, under deep anesthesia by ketamine/xylazine. No animal was exposed to euthanasia of another animal or handling of animals’ carcasses.

### Quantitative real-time PCR

RT-qPCR was performed to quantify LGTV RNA from in vitro and in vivo experiments to evaluate LGTV growth kinetics and viremia, respectively. For viremia, the blood samples (*n* = 3) from each group collected at 1, 3, 6, 24, 48, 72 h, were pooled before RNA extraction. Total RNA was extracted using QIAamp Viral RNA Mini Kit (Qiagen, Hilden, Germany). cDNA was synthesized using a SuperScript™ IV VILO™ Master Mix (Invitrogen, Thermo Fisher Scientific, Waltham, MA, USA). RT-qPCR was performed using Applied Biosystems products (Thermo Fisher Scientific, Waltham, MA, USA), including TaqMan Fast Advanced Master Mix, Custom TaqMan Gene Expression Assays and a QuantStudio 7 Flex Real-Time PCR System, as described previously^44^. A standard curve was used to calculate RNA copies/mL of LGTV. The standard curve was generated by plotting the serial dilutions of a known quantity of LGTV RNA against the cycle threshold (Ct) values of the samples.

### Enzyme-linked immunosorbent assay

Enzyme-linked immunosorbent assay (ELISA) was performed using mice serum samples collected 21 dpi to measure anti-TBEV IgG levels using the IMMUNOZYM® FSME (TBE) IgG All Species (PROGEN Biotechnik GmbH, Heidelberg, Germany). The experiments were performed in duplicates, and samples were analyzed according to the manufacturer’s instructions. Absorbance was read using Epoch 2 microplate spectrophotometer (BioTEK, Bad Friedrichshall, Germany). Sample concentrations were calculated in VIEU/mL using standard curve, and a threshold of >126 VIEU/mL was set to define positive samples.

### Immunofluorescence

Vero E6 cells were seeded into T25 flaks. Cells at approximately 80% confluency were inoculated with the rescued LGTV ICs. The inoculum was replaced with 2% HI–FBS medium after 2 h to remove the unbound virus particles, and the cells were further incubated at 37 °C. The cells were fixed at 3 dpi using methanol for 20 min, permeabilized with 0.5% Triton X-100 (Sigma-Aldrich, St. Louis, MO, USA) and blocked with 3% bovine serum albumin (Fitzgerald, Biosynth, Staad, Switzerland) in PBS. Mouse monoclonal anti-TBEV E primary antibody (United States Army Medical Research, Institute of Infectious Diseases, Fort Detrick, Frederick, MD, USA) and Alexa 488 secondary antibody (Invitrogen, Thermo Fisher Scientific, Waltham, MA, USA) were used for immunofluorescent labeling of LGTV. DAPI (Sigma-Aldrich, St. Louis, MO, USA) was used to stain cell nuclei. Images were captured using an Olympus BX41 microscope mounted with Olympus UC90 camera connected with Olympus CellSense standard 1.18 imaging software (Olympus, Vienna, Austria).

### Immunoblotting

Immunoblotting was performed using cell culture media collected 7 dpt. The media was centrifuged at 4000 *g* for 10 min to remove cellular debris and boiled in 4× LDS buffer (Invitrogen, Thermo Fisher Scientific, Waltham, MA, USA) for 5 min. Proteins were separated on pre-cast 4–12% polyacrylamide Bis-Tris gels (Invitrogen, Thermo Fisher Scientific, Waltham, MA, USA) before being transferred to nitrocellulose membranes using the iBlot 2 Gel Transfer Device (Invitrogen, Thermo Fisher Scientific, Waltham, MA, USA). Detection was performed using monoclonal mouse anti-LGTV E antibody (Clone 11H12, United States Army Medical Research, Institute of Infectious Diseases, Fort Detrick, Frederick, MD, USA).

### Statistical analysis

Statistical analysis for data from in vitro and in vivo experiments was carried out using GraphPad Prism (version 10.0.0, GraphPad Software Inc.). An unpaired Student *t*-test was performed for single comparison and a nonparametric ordinary one-way ANOVA was used for multiple comparisons. When significant effects were observed in the ANOVA, subsequent pairwise comparisons between experimental groups were performed using Tukey’s test. Body weight data was analyzed using a two-way ANOVA, followed by Dunnett’s post-hoc test to compare LGTV_DNA+L–2000_ and LGTV_Lab_ groups with the control group. A *p*-value < 0.05 was considered statistically significant.

## Supplementary information

Supplementary information

## Data Availability

Next generation sequencing data supporting the findings of this study have been deposited as unassembled FASTQ files at NCBI Sequence Read Archive under BioProject ID: PRJNA1259924 with BioSample accessions: SAMN48381464–SAMN48381471.

## References

[1] Kwasnik, M., Rola, J. & Rozek, W. Tick-borne encephalitis-review of the current status. *J. Clin. Med.* **12**, 6603 (2023). 37892741 10.3390/jcm12206603 PMC10607749

[2] Angulo, F. J. et al. Publicly available surveillance data on tick-borne encephalitis in Europe, 2023. *Ticks Tick. Borne Dis.* **15**, 102388 (2024). 39137541 10.1016/j.ttbdis.2024.102388

[3] Van Heuverswyn, J. et al. Spatiotemporal spread of tick-borne encephalitis in the EU/EEA, 2012 to 2020. *Eur. Surveill.* **28**, 2200543 (2023). 10.2807/1560-7917.ES.2023.28.11.2200543 PMC10021474 36927718

[4] Kubinski, M. et al. Tick-borne Encephalitis virus: a quest for better vaccines against a virus on the rise. *Vaccines* **8**, 451 (2020). 32806696 10.3390/vaccines8030451 PMC7564546

[5] Hansson, K. E. et al. Tick-borne Encephalitis vaccine failures: a 10-year retrospective study supporting the rationale for adding an extra priming dose in individuals starting at age 50 years. *Clin. Infect. Dis.* **70**, 245–251 (2020). 30843030 10.1093/cid/ciz176 PMC6938976

[6] Dutta, S. K. & Langenburg, T. A perspective on current flavivirus vaccine development: a brief review. *Viruses* **15**, 860 (2023). 37112840 10.3390/v15040860 PMC10142581

[7] Gritsun, T. S., Lashkevich, V. A. & Gould, E. A. Tick-borne encephalitis. *Antivir. Res.* **57**, 129–146 (2003). 12615309 10.1016/s0166-3542(02)00206-1

[8] Ruzek, D. et al. Tick-borne encephalitis in Europe and Russia: review of pathogenesis, clinical features, therapy, and vaccines. *Antivir. Res.* **164**, 23–51 (2019). 30710567 10.1016/j.antiviral.2019.01.014

[9] Pletnev, A. G. et al. Chimeric Langat/Dengue viruses protect mice from heterologous challenge with the highly virulent strains of tick-borne encephalitis virus. *Virology* **274**, 26–31 (2000). 10936085 10.1006/viro.2000.0426

[10] Rumyantsev, A. A., Murphy, B. R. & Pletnev, A. G. A tick-borne Langat virus mutant that is temperature sensitive and host range restricted in neuroblastoma cells and lacks neuroinvasiveness for immunodeficient mice. *J. Virol.* **80**, 1427–1439 (2006). 16415020 10.1128/JVI.80.3.1427-1439.2006 PMC1346960

[11] Thind, I. S. & Price, W. H. A chick embryo attenuated strain (TP21 E5) of Langat virus. I. Virulence of the virus for mice and monkeys. *Am. J. Epidemiol.* **84**, 193–213 (1966). 4958356 10.1093/oxfordjournals.aje.a120633

[12] Lindqvist, R. et al. The envelope protein of tick-borne encephalitis virus influences neuron entry, pathogenicity, and vaccine protection. *J. Neuroinflamm.* **17**, 284 (2020). 10.1186/s12974-020-01943-w PMC7523050 32988388

[13] Aubry, F. et al. Single-stranded positive-sense RNA viruses generated in days using infectious subgenomic amplicons. *J. Gen. Virol.* **95**, 2462–2467 (2014). 25053561 10.1099/vir.0.068023-0 PMC4202267

[14] Nogales, A. & Martinez-Sobrido, L. Reverse genetics approaches for the development of influenza vaccines. *Int. J. Mol. Sci.* **18**, 20 (2016). 28025504 10.3390/ijms18010020 PMC5297655

[15] Yeh, M. T. et al. Engineering the live-attenuated polio vaccine to prevent reversion to virulence. *Cell Host Microbe* **27**, 736–751.e8 (2020). 32330425 10.1016/j.chom.2020.04.003 PMC7566161

[16] Li, G., Teleki, C. & Wang, T. Memory T cells in flavivirus vaccination. *Vaccines* **6**, 73 (2018). 30340377 10.3390/vaccines6040073 PMC6313919

[17] Wu, B., Qi, Z. & Qian, X. Recent advancements in mosquito-borne flavivirus vaccine development. *Viruses* **15**, 813 (2023). 37112794 10.3390/v15040813 PMC10143207

[18] Mansfield, K. L. et al. Tick-borne encephalitis virus - a review of an emerging zoonosis. *J. Gen. Virol.* **90**, 1781–1794 (2009). 19420159 10.1099/vir.0.011437-0

[19] Aubry, F. et al. Flavivirus reverse genetic systems, construction techniques and applications: a historical perspective. *Antivir. Res.* **114**, 67–85 (2015). 25512228 10.1016/j.antiviral.2014.12.007 PMC7173292

[20] Pu, S. Y. et al. Successful propagation of flavivirus infectious cDNAs by a novel method to reduce the cryptic bacterial promoter activity of virus genomes. *J. Virol.* **85**, 2927–2941 (2011). 21228244 10.1128/JVI.01986-10 PMC3067970

[21] Asghar, N. et al. The role of the poly(A) tract in the replication and virulence of tick-borne encephalitis virus. *Sci. Rep.* **6**, 39265 (2016). 27982069 10.1038/srep39265 PMC5159820

[22] Helmova, R. et al. Tick-borne Encephalitis virus adaptation in different host environments and existence of quasispecies. *Viruses* **12**, 902 (2020). 32824843 10.3390/v12080902 PMC7472235

[23] Litov, A. G. et al. Evaluation of the population heterogeneity of TBEV laboratory variants using high-throughput sequencing. *J. Gen. Virol.* **99**, 240–245 (2018). 29393021 10.1099/jgv.0.001003

[24] Emeny, J. M. & Morgan, M. J. Regulation of the interferon system - evidence that vero cells have a genetic defect in interferon-production. *J. Gen. Virol.* **43**, 247–252 (1979). 113494 10.1099/0022-1317-43-1-247

[25] Frumence, E. et al. The South Pacific epidemic strain of Zika virus replicates efficiently in human epithelial A549 cells leading to IFN-beta production and apoptosis induction. *Virology* **493**, 217–226 (2016). 27060565 10.1016/j.virol.2016.03.006

[26] Lauring, A. S., Jones, J. O. & Andino, R. Rationalizing the development of live attenuated virus vaccines. *Nat. Biotechnol.* **28**, 573–579 (2010). 20531338 10.1038/nbt.1635 PMC2883798

[27] Nouveau, L. et al. Immunological analysis of the murine anti-CD3-induced cytokine release syndrome model and therapeutic efficacy of anti-cytokine antibodies. *Eur. J. Immunol.* **51**, 2074–2085 (2021). 33945643 10.1002/eji.202149181 PMC8237068

[28] Li, L. et al. Attenuation of Zika virus by passage in human hela cells. *Vaccines* **7**, 93 (2019). 31434319 10.3390/vaccines7030093 PMC6789458

[29] Resch, T. K. et al. Serial passaging of the human rotavirus CDC-9 strain in cell culture leads to attenuation: characterization from in vitro and in vivo studies. *J. Virol.* **94**, e00889–20 (2020). 32461318 10.1128/JVI.00889-20 PMC7375378

[30] Zhang, L. et al. Serial cell culture passaging in vitro led to complete attenuation and changes in the characteristic features of a virulent porcine deltacoronavirus strain. *J. Virol.* **98**, e0064524 (2024). 39012141 10.1128/jvi.00645-24 PMC11334472

[31] Hervé, C. et al. The how’s and what’s of vaccine reactogenicity. *Npj Vaccines* **4**, 39 (2019). 31583123 10.1038/s41541-019-0132-6 PMC6760227

[32] Ahmed, W. et al. An experimental murine model to study acquisition dynamics of tick-borne langat virus in ixodes scapularis. *Front. Microbiol.* **13**, 849313 (2022). 35495703 10.3389/fmicb.2022.849313 PMC9048798

[33] Barrett, A. D. T. Yellow fever live attenuated vaccine: a very successful live attenuated vaccine but still we have problems controlling the disease. *Vaccine* **35**, 5951–5955 (2017). 28366605 10.1016/j.vaccine.2017.03.032

[34] Lobigs, M. et al. Live chimeric and inactivated japanese Encephalitis virus vaccines differ in their cross-protective values against murray valley Encephalitis virus. *J. Virol.* **83**, 2436–2445 (2009). 19109382 10.1128/JVI.02273-08 PMC2648276

[35] Rivera, L. et al. Three-year efficacy and safety of Takeda’s Dengue vaccine candidate (TAK-003). *Clin. Infect. Dis.* **75**, 107–117 (2022). 34606595 10.1093/cid/ciab864 PMC9402653

[36] Kubinski, M. et al. Cross-reactive antibodies against Langat virus protect mice from lethal tick-borne encephalitis virus infection. *Front. Immunol.* **14**, 1134371 (2023). 36926332 10.3389/fimmu.2023.1134371 PMC10011100

[37] Petry, M. et al. Immunity to TBEV related flaviviruses with reduced pathogenicity protects mice from disease but not from TBEV entry into the CNS. *Vaccines* **9**, 196 (2021). 33652698 10.3390/vaccines9030196 PMC7996866

[38] Ruzek, D. et al. CD8+ T-cells mediate immunopathology in tick-borne encephalitis. *Virology* **384**, 1–6 (2009). 19070884 10.1016/j.virol.2008.11.023

[39] Smith, C. E. A virus resembling Russian spring-summer encephalitis virus from an ixodid tick in Malaya. *Nature* **178**, 581–582 (1956). 13369466 10.1038/178581a0

[40] Bolger, A. M., Lohse, M. & Usadel, B. Trimmomatic: a flexible trimmer for Illumina sequence data. *Bioinformatics.* **30**, 2114–2120 (2014). 10.1093/bioinformatics/btu170 PMC4103590 24695404

[41] Li, H. & Durbin, R. Fast and accurate short read alignment with Burrows-Wheeler transform. *Bioinformatics.* **25**, 1754–1760 (2009). 10.1093/bioinformatics/btp324 PMC2705234 19451168

[42] Wilm, A. et al. LoFreq: a sequence-quality aware, ultra-sensitive variant caller for uncovering cell-population heterogeneity from high-throughput sequencing datasets. *Nucleic Acids Res.* **40**, 11189–11201 (2012). 10.1093/nar/gks918 PMC3526318 23066108

[43] Berglund, E. C. et al. Accurate detection of subclonal single nucleotide variants in whole genome amplified and pooled cancer samples using HaloPlex target enrichment. *BMC Genomics.* **14**, 856 (2013). 10.1186/1471-2164-14-856 PMC4046713 24314227

[44] Tran, P. T. et al. Roles of the endogenous lunapark protein during flavivirus replication. *Viruses* **13**, 1198 (2021). 34206552 10.3390/v13071198 PMC8310331

[45] Hoornweg, T. E. et al. Rescue and in vitro characterization of a divergent TBEV-Eu strain from the Netherlands. *Sci. Rep.* **13**, 2872 (2023). 36807371 10.1038/s41598-023-29075-0 PMC9938877

