# Supplementary material for: Development of Langat virus infectious clones as a platform for live-attenuated tick-borne encephalitis vaccine
Source: Npj Viruses. 2025 May 23;3:44. doi: 10.1038/s44298-025-00129-6 (PMC12102213; doi:10.1038/s44298-025-00129-6)
Supplement: Supplementary file 1 — Supplementary information [file 44298_2025_129_MOESM1_ESM.pdf]

Supplementary Table 1. List of single nucleotide variants (SNVs) identified using lofreq with at least one sample having SNV fraction > 1%. Sequencing depth of each position is indicated in parentheses.

				Passage 0				Passage 5			
Position	Mutation	Region	A.A. change	RNA+L-Max	RNA+L-2000	DNA+L-2000	DNA+Amaxa	RNA+L-Max	RNA+L-2000	DNA+L-2000	DNA+Amaxa
131	A to T	C	M1L	0 (1262)	0.3 (2108)	1.69 (3599)	0.09 (2313)	0.05 (2176)	0 (2282)	0 (2198)	0 (1430)
403	C to A	C	R91R	1.59 (2496)	70.87 (3802)	0.16 (6577)	0.05 (4421)	0 (4131)	0.17 (4258)	0.03 (4121)	0 (2606)
647	G to A	prM	E173K	0.04 (2612)	0 (4136)	2.04 (6640)	0.02 (4764)	0 (4500)	0 (4864)	0 (4550)	0 (2917)
1408	C to A	E	H426Q	0.8 (1434)	0.95 (1670)	0.3 (2748)	1.2 (2322)	1.2 (2199)	1.17 (2368)	0.91 (2240)	0.65 (1875)
1412	G to C	E	G428R	0.8 (1414)	0.91 (1608)	0.31 (2615)	1.25 (2292)	1.11 (2188)	1.06 (2394)	0.96 (2220)	0.47 (1949)
1413	G to T	E	G428V	0.73 (1413)	0.91 (1608)	0.31 (2614)	1.13 (2292)	1.11 (2187)	1.07 (2396)	0.92 (2219)	0.42 (1948)
1414	A to C	E	G428G	0.81 (1398)	0.94 (1579)	0.32 (2564)	1.3 (2250)	1.13 (2156)	1.1 (2352)	0.94 (2193)	0.49 (1926)
1415	G to C	E	E429Q	0.73 (1402)	0.94 (1584)	0.32 (2567)	1.19 (2255)	1.12 (2166)	1.08 (2364)	0.92 (2197)	0.47 (1936)
1419	T to G	E	F430C	0.84 (1337)	0.9 (1506)	0.38 (2412)	1.39 (2145)	1.29 (2041)	1.17 (2252)	1.03 (2068)	0.43 (1908)
1561	T to C	E	T477T	1.22 (2021)	0 (1971)	0.04 (2915)	0.06 (3307)	0.03 (3274)	0 (3792)	0.03 (3252)	0.05 (4227)
1584	C to A	E	T485K	0 (2153)	0 (2045)	1.08 (3129)	0 (3463)	0 (3475)	0 (4036)	0 (3395)	0 (4468)
1696	G to A	E	V522V	0 (2328)	0 (2293)	0.03 (3406)	7.65 (3975)	0.03 (3928)	0.02 (4522)	0 (3863)	0.04 (4955)
1800	A to G	E	E557G	0.14 (2287)	14.8 (2337)	1.8 (3410)	0.05 (3897)	0.03 (3907)	0.05 (4456)	0.05 (3896)	0 (5006)
1802	G to A	E	G558R	0.05 (2270)	0 (2318)	1.66 (3381)	0.03 (3862)	0.03 (3875)	0 (4417)	0 (3873)	0 (4952)
2034	C to T	E	A635V	0.05 (2132)	0 (2150)	2.19 (3220)	0 (3637)	0 (3882)	0 (4260)	0.06 (3773)	0 (4790)
2068	C to T	E	N646N	0 (2099)	0 (2123)	0 (3148)	0 (3512)	0 (3889)	2.54 (4073)	0.03 (3802)	0.04 (4645)
2142	A to G	E	Q671R	0 (2031)	0 (1909)	0.07 (2940)	0.03 (3013)	0.84 (3530)	0.09 (3533)	19.69 (3579)	0.48 (4033)
2192	A to G	E	K688E	0.05 (2103)	0 (1951)	0.07 (2942)	0.03 (3087)	18.55 (3642)	97.87 (3588)	1.74 (3476)	96.34 (4025)
2205	G to C	E	R692T	0 (2111)	0 (1976)	0 (2967)	0 (3119)	0.37 (3865)	0 (3703)	11.15 (3936)	0.2 (4133)
2205	G to T	E	R692I	0 (2111)	0 (1976)	0.03 (2967)	0.03 (3119)	40.07 (3865)	0.94 (3703)	58.96 (3936)	1.48 (4133)
2206	A to T	E	R692S	0 (2108)	0.05 (1970)	0 (2949)	0 (3116)	0.05 (3868)	0 (3689)	3.84 (3929)	0.1 (4110)
2216	C to T	E	L696F	0 (2206)	0 (2055)	0 (3063)	0.1 (3236)	13.1 (3907)	0.24 (3810)	0.33 (3734)	0.05 (4261)
2229	C to A	E	A700D	0 (2172)	0 (2005)	0 (3012)	0.03 (3163)	7.45 (3791)	0.25 (3768)	0.22 (3706)	0.12 (4215)

2282	C to T	E	H718Y	0.55 (2099)	0.16 (1961)	0.07 (2899)	0.03 (3097)	5.78 (3708)	0.32 (3611)	3.24 (3544)	0.73 (4129)
2318	G to T	E	G730W	0 (1999)	0 (1893)	0.04 (2803)	0.04 (2942)	13.18 (3934)	0.26 (3649)	0.06 (3524)	0.02 (4122)
2429	G to A	E	V767M	0 (2125)	0 (2069)	0 (3167)	0.07 (3180)	16.68 (4068)	0.45 (3913)	0.14 (3645)	0.07 (4397)
2440	G to A	E	M770I	1.05 (2050)	0 (1967)	0 (3051)	0.84 (3071)	0 (3823)	0 (3780)	0 (3480)	0 (4259)
2442	C to T	E	T771I	0 (2111)	3.75 (2025)	0.2 (3120)	0 (3171)	0.03 (3920)	0.03 (3894)	0 (3585)	0 (4395)
3267	C to T	NS1	A1046V	1.8 (2913)	0.14 (3746)	0.02 (4680)	0.04 (5343)	0.03 (3092)	0.03 (3614)	0.02 (4163)	0.05 (4405)
3269	C to T	NS1	H1047Y	0 (2891)	0 (3729)	0.05 (4648)	0.28 (5322)	0.07 (3077)	0.06 (3591)	1.68 (4128)	0.09 (4384)
3631	C to T	NS2A	G1167G	1.81 (2778)	0.14 (3740)	0.02 (4887)	0.1 (5314)	0.03 (3368)	0.03 (3960)	0 (4496)	0.04 (4829)
3665	G to A	NS2A	V1179I	0 (2996)	28.63 (4071)	0.55 (5128)	0.09 (5606)	0.14 (3551)	94.7 (4211)	0.54 (4704)	0.43 (5023)
3700	A to G	NS2A	A1190A	4.15 (3029)	0.08 (4035)	0.02 (5136)	0 (5635)	0.06 (3458)	0.03 (4100)	0 (4645)	0 (4934)
3775	G to A	NS2A	M1215I	0 (3097)	1.3 (4187)	0.02 (5389)	0.02 (5755)	0 (3398)	0 (4163)	0 (4723)	0 (4932)
3938	T to C	NS2A	F1270L	0 (5639)	0.02 (6467)	0.02 (5959)	0.02 (8562)	0.07 (5840)	0.11 (6232)	8.18 (8114)	0.25 (8290)
3972	T to C	NS2A	I1281T	0 (6044)	0 (6908)	0 (5953)	0.01 (9004)	1.7 (6273)	0.12 (6580)	0.13 (8556)	0.17 (8833)
4030	C to T	NS2A	G1300G	0 (6585)	3.04 (7360)	0.04 (5747)	0.33 (9503)	0.03 (6785)	0.06 (6911)	0.01 (9117)	0.04 (9279)
4062	T to C	NS2A	M1311T	0.02 (6482)	0.03 (7223)	0 (5558)	0.01 (9314)	0 (6702)	0.03 (6831)	1.93 (8939)	0.07 (9096)
4153	A to G	NS2A	S1341S	5.56 (6809)	0.2 (7291)	0.08 (5113)	0.01 (9118)	0.04 (6996)	0.05 (6752)	0.02 (9037)	0.02 (9418)
4299	C to T	NS2B	A1390V	0.05 (6287)	0.05 (6144)	0.03 (3398)	0.08 (7753)	13.22 (6155)	5.13 (5625)	6.14 (7827)	37.2 (8531)
4325	C to T	NS2B	L1399F	0 (6203)	0.02 (6033)	0 (3075)	0.01 (7469)	0.1 (5998)	2.71 (5440)	8.59 (7624)	0.49 (8236)
4343	A to G	NS2B	K1405E	0.02 (6061)	0.02 (5828)	0 (2748)	1.59 (7162)	0 (5821)	0 (5210)	0.03 (7246)	0.01 (7931)
4682	A to G	NS3	T1518A	0 (5116)	0.26 (4649)	0 (882)	0 (5379)	10.41 (4853)	1.14 (3810)	0.86 (6212)	0.33 (6409)
4720	G to T	NS3	V1530V	0 (4475)	0.03 (3990)	0 (723)	0.02 (4654)	79.03 (4177)	0.27 (3119)	0.1 (5266)	0.04 (5317)
4816	T to G	NS3	A1562A	0 (4977)	3.58 (4737)	0 (888)	0 (5603)	0 (4607)	0 (3651)	0 (6352)	0 (6399)
4858	T to C	NS3	S1576S	0 (4598)	0.02 (4347)	0 (804)	0 (5167)	0.02 (4376)	0.06 (3426)	1.46 (5908)	0.07 (6086)
5276	T to G	NS3	L1716V	0 (5091)	1.1 (4812)	0 (834)	0 (5630)	0 (4967)	0 (4210)	0 (6362)	0 (6722)
5353	A to G	NS3	A1741A	0 (4890)	0.02 (4538)	3.78 (822)	0.02 (5245)	0 (4693)	0.05 (3889)	0 (6071)	0.02 (6483)
5368	C to T	NS3	H1746H	0 (4922)	0 (4569)	0 (837)	0.02 (5294)	0.04 (4739)	0.05 (3922)	2.97 (6214)	0.17 (6582)
5620	G to A	NS3	E1830E	0 (3246)	1.8 (3046)	0.19 (548)	0.89 (3676)	0.09 (3338)	0 (2776)	0 (4354)	0.02 (4528)
5670	C to T	NS3	T1847I	0 (2662)	0 (2451)	0 (452)	3.84 (2983)	0.04 (2754)	0 (2304)	0 (3572)	0.06 (3745)

5775	G to T	NS3	S1882I	0.82 (1768)	0.71 (1634)	5.53 (936)	0.84 (1717)	0.18 (1660)	0 (1274)	0 (1723)	0 (1827)
6211	G to A	NS3	L2027L	0.09 (4348)	0.03 (3522)	1.04 (7042)	0 (4182)	0.06 (3551)	0 (1535)	0 (346)	0 (685)
6232	A to C	NS3	T2034T	9.02 (4432)	0.06 (3578)	0 (7157)	0 (4223)	16.59 (3616)	0.07 (1575)	0.28 (365)	0.15 (682)
6499	A to T	NS4A	P2123P	52.2 (4499)	0.27 (3465)	0.13 (6604)	0.18 (4015)	77.33 (3401)	0.61 (1508)	0 (354)	0.3 (670)
6562	G to A	NS4A	E2144E	52.78 (4415)	0.35 (3344)	0.05 (6627)	0.24 (3863)	77.5 (3440)	0.85 (1470)	0.55 (371)	0.15 (702)
6702	T to C	NS4A	V2191A	0 (3785)	0 (3019)	0.02 (5689)	0.03 (3297)	0 (3034)	4.67 (1374)	0.3 (354)	0.91 (575)
6768	A to C	NS4A	N2213T	0 (3510)	0 (2901)	0 (5507)	0 (3147)	0.77 (3025)	3.84 (1371)	0 (314)	0 (552)
6840	G to C	NS4A	S2237T	0 (3772)	0 (3160)	0 (6218)	0 (3503)	1.6 (3379)	0 (1503)	0 (364)	0 (648)
6884	G to A	NS4A	G2252S	0.06 (3304)	0 (2852)	0.06 (5571)	0.07 (3208)	0.17 (2944)	0.77 (1338)	0 (308)	17.43 (589)
7156	A to G	NS4B	G2342G	1.11 (2231)	0.6 (1749)	1.35 (3493)	1.39 (2195)	0.34 (1821)	0.75 (824)	1.14 (181)	0.27 (377)
7260	C to T	NS4B	A2377V	3.63 (1730)	0 (1415)	0 (2381)	0 (1462)	0 (1506)	0 (791)	0 (273)	0 (401)
7516	T to C	NS4B	A2462A	0.08 (2741)	0.09 (2347)	1.63 (469)	0 (458)	0.04 (2734)	0 (2633)	0 (1573)	0 (1522)
7753	T to C	NS5	A2541A	0.07 (3004)	2.08 (2367)	0.19 (554)	0.2 (527)	0 (2978)	0 (2899)	0.06 (1729)	0.19 (1643)
7972	C to T	NS5	Y2614Y	0.03 (4080)	0.03 (3213)	0 (687)	4.07 (655)	0 (3698)	0 (3468)	0 (2193)	0 (2103)
8048	G to T	NS5	G2640C	0.05 (4080)	2.74 (3185)	0 (707)	0 (667)	0 (3795)	0 (3519)	0 (2158)	0 (2200)
8093	C to T	NS5	L2655L	0 (4057)	0.03 (3190)	0 (737)	0.16 (654)	19.44 (4037)	0.14 (3670)	0 (2256)	0.04 (2277)
8203	T to G	NS5	C2691W	0 (3380)	0 (2836)	0 (595)	3.55 (574)	0 (3611)	0 (3292)	0 (2012)	0 (2032)
8321	A to G	NS5	M2731V	0 (3093)	0 (2651)	0.18 (579)	4.79 (580)	0.03 (3387)	0 (3106)	0 (1848)	0.05 (1944)
8338	C to T	NS5	A2736A	0 (3035)	0 (2656)	0 (557)	0 (587)	0 (3344)	0 (3082)	1.86 (1806)	0.05 (1918)
8376	C to A	NS5	S2749Y	0 (2888)	2.41 (2526)	0 (525)	0 (537)	0 (3152)	0 (2959)	0 (1713)	0 (1844)
8993	T to C	NS5	L2955L	1.19 (3066)	0 (1849)	0 (1653)	0 (2382)	0.08 (2624)	0.06 (3273)	0.04 (2456)	0.06 (3187)
9251	C to T	NS5	L3041L	1.44 (3731)	0 (2120)	0 (2208)	0 (2775)	0 (2883)	0 (3461)	0 (2879)	0 (3564)
9402	G to A	NS5	R3091K	0 (4620)	0 (2596)	0 (2774)	0 (3391)	0 (3109)	0 (4016)	0 (3507)	1.25 (4100)
9419	C to G	NS5	R3097G	1.05 (4697)	0.29 (2605)	0 (2843)	0 (3453)	0.07 (3154)	0.03 (4010)	0 (3549)	0.02 (4159)
9727	C to T	NS5	T3199T	16.32 (4834)	12.47 (2425)	48.86 (3846)	6.26 (2832)	25.8 (5239)	26.67 (6370)	34.16 (6272)	30.7 (6712)
9734	G to A	NS5	D3202N	21.51 (5126)	13.17 (2455)	53.88 (3997)	7.48 (2866)	33.86 (5809)	34.22 (7002)	60.09 (7138)	38.86 (7475)
9878	G to A	NS5	A3250T	0 (5346)	1.3 (2386)	0.05 (4136)	0 (2894)	0.02 (6415)	0 (7675)	0.01 (7801)	0.03 (8062)
10019	G to A	NS5	V3297M	0.19 (6312)	33.03 (3249)	1.63 (3784)	0.12 (3463)	0.1 (5959)	0.31 (7273)	0.09 (7662)	0.03 (7737)

10036	A to C	NS5	T3302T	0.07 (6176)	1.37 (3199)	2.54 (3615)	0.89 (3382)	0 (5814)	0.01 (7099)	0.05 (7555)	0.03 (7619)
10041	G to A	NS5	R3304K	0.08 (6201)	1.65 (3231)	3.26 (3604)	1.18 (3389)	0.05 (5765)	0.07 (7038)	0.18 (7488)	0.09 (7579)
10046	A to C	NS5	T3306P	0.02 (6357)	0.93 (3302)	1.33 (3654)	0.36 (3461)	0.02 (5820)	0 (7125)	0.04 (7560)	0.04 (7625)
10226	G to A	NS5	G3366S	0.08 (5201)	0 (2899)	0 (2757)	0 (2764)	0.03 (3942)	0.02 (4890)	1.15 (5799)	0.06 (5409)
10370	T to A	NS5	F3414I	0.66 (4557)	0 (2813)	0.56 (2410)	0 (2706)	0.03 (3432)	0.05 (4293)	0.02 (4711)	8.14 (4689)
10370	T to C	NS5	F3414L	0.41 (4557)	0 (2813)	0 (2410)	0.04 (2706)	0.95 (3432)	1.22 (4293)	0.35 (4711)	0.44 (4689)
10513	C to T	3´NCR	.	2.32 (3814)	0.09 (2456)	0 (1994)	0 (2178)	0 (2763)	0.03 (3400)	0.05 (3807)	0.03 (3623)
10561	A to G	3´NCR	.	0.03 (3587)	9.6 (2444)	0.32 (1940)	0 (2093)	0 (2622)	0 (3157)	0 (3543)	0 (3371)
10567	G to A	3´NCR	.	0.12 (3399)	0 (2243)	0 (1847)	1.76 (2021)	0.12 (2508)	0 (3007)	0.03 (3379)	0 (3192)
10614	T to C	3´NCR	.	0 (3413)	3.33 (2179)	0.12 (1799)	0 (2005)	0.08 (2539)	0.07 (2902)	0.1 (3259)	0.03 (3120)
10767	G to T	3´NCR	.	13.8 (2708)	16.16 (1764)	0.67 (1386)	0.06 (1601)	0.11 (1873)	95.82 (1865)	1.44 (2622)	0.21 (2445)
10768	A to T	3´NCR	.	13.67 (2680)	16.29 (1753)	0.68 (1370)	0 (1582)	0.11 (1855)	96.09 (1858)	1.46 (2597)	0.21 (2425)
10769	A to T	3´NCR	.	13.44 (2663)	16.34 (1744)	0.54 (1360)	0 (1570)	0.11 (1843)	95.86 (1848)	1.43 (2585)	0.17 (2403)
10770	A to C	3´NCR	.	14.11 (2692)	16.84 (1753)	0.7 (1381)	0 (1585)	0.11 (1851)	96.11 (1870)	1.5 (2606)	0.17 (2424)
10811	A to T	3´NCR	.	0.04 (2308)	0 (1337)	0 (1137)	2.04 (1273)	0 (1485)	0 (1783)	0 (2129)	0 (2021)
10905	T to G	3´NCR	.	0 (516)	0 (317)	0 (214)	5.96 (243)	0 (225)	0 (284)	0 (363)	0 (328)
10913	A to T	3´NCR	.	2.48 (247)	3.4 (155)	0.75 (138)	0 (140)	0.71 (142)	0 (194)	0 (252)	0 (212)
10921	C to T	3´NCR	.	1.39 (149)	1.25 (91)	0 (87)	0 (78)	0 (72)	1.04 (102)	3.76 (143)	0.87 (121)

Supplementary Note 1. General health assessment of mice recorded at different time points and descriptive analyses.

A. Body Weights – Individual Values

Absolute body weights (g) following intramuscular injection of $LGTV_{Lab}$ (Group 1), $LGTV_{DNA+L–2000}$ (Group 2) or Mock (Group 3). Injection was performed on Day 0.

Group	Mice ID	Day 0	Day 1	Day 2	Day 3	Day 7	Day 10	Day 14	Day 17	Day 21
1	550	19.6	19.9	20.1	20.0	21.3	20.7	22.0	22.0	22.7
	551	21.1	20.7	20.8	21.3	21.2	21.4	21.6	22.0	23.4
	552	20.6	20.8	21.1	21.1	21.4	22.3	22.7	21.9	23.6
	553	19.0	18.8	19.2	18.8	19.0	19.9	20.2	21.4	21.3
	554	19.4	19.3	20.0	19.7	20.1	19.9	21.6	21.2	21.8
	555	19.8	20.1	20.6	20.5	20.3	20.5	20.9	21.2	22.1
2	556	20.0	19.5	19.7	19.7	20.4	20.0	20.5	19.9	19.3
	557	21.5	21.3	21.1	21.1	21.6	22.1	22.8	22.8	23.2
	558	21.7	21.6	21.9	21.7	22.4	22.1	22.8	24.2	24.0
	559	21.8	21.1	20.6	20.7	21.6	21.5	21.7	22.3	22.7
	560	18.5	18.9	18.7	18.7	19.8	19.2	19.7	19.7	20.4
	561	19.2	19.2	19.0	18.6	20.0	20.3	20.9	21.1	21.4
3	562	18.9	19.3	19.6	19.6	20.1	20.0	21.4	21.6	22.0
	563	19.8	20.3	20.1	20.1	20.2	20.2	20.9	21.3	21.8
	564	20.1	20.5	20.2	20.1	20.8	20.1	20.6	20.9	21.3
	565	19.6	20.0	20.7	20.2	20.6	19.9	21.5	21.4	21.3
	566	19.2	19.1	19.3	18.8	19.0	18.9	19.7	20.6	21.1
	567	18.8	19.4	20.2	18.6	19.3	19.8	20.6	20.8	21.4

B. Absolute Body weight – Group statistics

Descriptive statistics of absolute body weights (g), showing mean, standard error of the mean (SEM) and number of animals (N).

Day	Group 1: $LGTV_{Lab}$			Group 2: $LGTV_{DNA+L–2000}$			Group 3: Mock		
	Mean	*SEM*	*N*	*Mean*	*SEM*	*N*	*Mean*	*SEM*	*N*
Day 0	19.92	0.32	6	20.45	0.58	6	19.40	0.21	6
Day 1	19.93	0.32	6	20.27	0.49	6	19.77	0.24	6
Day 2	20.30	0.28	6	20.17	0.51	6	20.02	0.20	6
Day 3	20.23	0.38	6	20.08	0.53	6	19.57	0.29	6
Day 7	20.55	0.38	6	20.97	0.43	6	20.00	0.29	6
Day 10	20.78	0.38	6	20.87	0.49	6	19.82	0.19	6
Day 14	21.50	0.35	6	21.40	0.52	6	20.78	0.27	6
Day 17	21.62	0.16	6	21.67	0.72	6	21.10	0.16	6
Day 21	22.48	0.37	6	21.83	0.73	6	21.48	0.14	6

C. Relative Body Weight - Group statistics

Descriptive statistics of relative body weights (%), showing mean, standard error of the mean (SEM) and number of animals (N). Data was analysed by two-way ANOVA, followed by a Dunnett's multiple comparison post-hoc test, comparing test item groups to Group 3 DMEM/HEPES. * p-value < 0.05.

Day	Group 1: $LGTV_{Lab}$			Group 2: $LGTV_{DNA+L\text{–}2000}$			Group 3: Mock		
	Mean	*SEM*	*N*	*Mean*	*SEM*	*N*	*Mean*	*SEM*	*N*
Day 0	100.00	0.00	6	100.00	0.00	6	100.00	0.00	6
Day 1	100.09	0.59	6	99.18	0.78	6	101.89	0.52	6
Day 2	101.96	0.78	6	98.68*	0.98	6	103.22	1.18	6
Day 3	101.57	0.63	6	98.26	0.89	6	100.86	0.94	6
Day 7	103.19	1.27	6	102.66	1.15	6	103.10	1.05	6
Day 10	104.36	0.99	6	102.13	1.05	6	102.19	1.19	6
Day 14	108.00	1.58	6	104.75	1.34	6	107.19	1.77	6
Day 17	108.63	1.37	6	105.96	1.84	6	108.83	1.42	6
Day 21	112.90	0.77	6	106.81*	2.33	6	110.81	1.52	6

D. Clinical Signs – Individual Findings

Clinical signs following intramuscular injection of $LGTV_{Lab}$ (Group 1), $LGTV_{DNA+L\text{–}2000}$ (Group 2) or Mock (Group 3). Injection was performed on Day 0. N/A: nothing adverse.

Group	ID	Day 0	Day 1	Day 2	Day 3	Day 7	Day 10	Day 14	Day 17	Day 21
1	550	N/A	N/A	N/A	N/A	N/A	N/A	N/A	N/A	N/A
	551	N/A	N/A	N/A	N/A	N/A	N/A	N/A	N/A	N/A
	552	N/A	N/A	N/A	N/A	N/A	N/A	N/A	N/A	N/A
	553	N/A	N/A	N/A	N/A	N/A	N/A	N/A	N/A	N/A
	554	N/A	N/A	N/A	N/A	N/A	N/A	N/A	N/A	N/A
	555	N/A	N/A	N/A	N/A	N/A	N/A	N/A	N/A	Slightly red in nose
2	556	N/A	N/A	Wound injection site	Wound injection site	Wound better	Wound red and irritated	N/A	N/A	Large wound on leg
	557	N/A	N/A	N/A	N/A	N/A	N/A	N/A	N/A	N/A
	558	N/A	N/A	N/A	N/A	N/A	N/A	N/A	N/A	N/A
	559	N/A	N/A	N/A	N/A	N/A	N/A	N/A	N/A	N/A
	560	N/A	N/A	N/A	N/A	N/A	N/A	N/A	N/A	N/A
	561	N/A	N/A	N/A	N/A	N/A	N/A	N/A	N/A	N/A
3	562	N/A	N/A	N/A	N/A	N/A	N/A	N/A	N/A	N/A
	563	N/A	N/A	N/A	N/A	N/A	N/A	N/A	N/A	N/A
	564	N/A	N/A	N/A	N/A	N/A	N/A	N/A	N/A	N/A
	565	N/A	N/A	N/A	N/A	N/A	N/A	N/A	N/A	N/A
	566	N/A	N/A	N/A	N/A	N/A	N/A	N/A	N/A	N/A
	567	N/A	N/A	N/A	N/A	N/A	N/A	N/A	N/A	N/A
